# Cladribine with cyclophosphamide and prednisone in the management of low-grade lymphoproliferative malignancies

**DOI:** 10.1038/sj.bjc.6690195

**Published:** 1999-03

**Authors:** F M Laurencet, G B Zulian, M Guetty-Alberto, P A Iten, D C Betticher, P Alberto

**Affiliations:** Division of Oncology, Geneva University Hospitals, Geneva, Switzerland; Division of Oncology, Bern University Hospital for the SAKK Lymphoma Group, Bern, Switzerland

## Abstract

The feasibility of combining cladribine with cyclophosphamide and prednisone in the management of indolent lymphoid malignancies was determined. Nineteen patients [nine chronic lymphocytic leukaemia (CLL), seven non-Hodgkin's lymphoma (NHL) and three macroglobulinaemia (M))] received cladribine 0.1 mg kg^−1^ per day as a subcutaneous bolus injection on days 1–3 (up to 5 injections) with intravenous cyclophosphamide 500 mg m^−2^ on day 1 and oral prednisone 40 mg m^−2^ on days 1–5 at 4-weekly intervals up to a maximum of six courses. A total of 80 courses were given. Overall response rate was 88%, with four patients achieving a complete clinical and haematological response and 12 achieving a partial response. Neutropenia WHO grade 4 in two patients and WHO grade 3 infection in one patient were the limiting toxicities on treatment. During the follow-up, WHO grade ≥3 haematological complications occurred in five patients and WHO grade ≥3 non-haematological complications in five patients. There were no treatment-related deaths. This study demonstrates the feasibility of the cladribine/cyclophosphamide/prednisone (CCP) combination that appears highly active and safe in the management of indolent lymphoid malignancies. © 1999 Cancer Research Campaign


*British Journal of Cancer* (1999) **79**(7/8), 1215–1219
© 1999 Cancer Research Campaign
Article no. bjoc.1998.0195

### Cladribine with cyclophosphamide and prednisone in the management of low-grade lymphoproliferative malignancies

**FM Laurencet[1], GB Zulian[1], M Guetty-Alberto[1], PA Iten[1], DC Betticher[2] and P Alberto[1]**

[1]Divisions of Oncology, Geneva University Hospitals and [2]Bern University Hospital for the SAKK Lymphoma Group, Switzerland

**Summary** The feasibility of combining cladribine with cyclophosphamide and prednisone in the management of indolent lymphoid malignancies was determined. Nineteen patients [nine chronic lymphocytic leukaemia (CLL), seven non-Hodgkin's lymphoma (NHL) and three macroglobulinaemia (M))] received cladribine 0.1 mg $kg^{-1}$ per day as a subcutaneous bolus injection on days 1–3 (up to 5 injections) with intravenous cyclophosphamide 500 mg $m^{-2}$ on day 1 and oral prednisone 40 mg $m^{-2}$ on days 1–5 at 4-weekly intervals up to a maximum of six courses. A total of 80 courses were given. Overall response rate was 88%, with four patients achieving a complete clinical and haematological response and 12 achieving a partial response. Neutropenia WHO grade 4 in two patients and WHO grade 3 infection in one patient were the limiting toxicities on treatment. During the follow-up, WHO grade ≥3 haematological complications occurred in five patients and WHO grade ≥3 non-haematological complications in five patients. There were no treatment-related deaths. This study demonstrates the feasibility of the cladribine/cyclophosphamide/prednisone (CCP) combination that appears highly active and safe in the management of indolent lymphoid malignancies.

Chronic lymphocytic leukaemia (CLL), non-Hodgkin's lymphoma CLL-like (NHL-A) and Waldenström's macroglobulinaemia (M) are incurable indolent B-cell lymphoproliferative disorders. Differences in response rate to various agents may be seen between these three distinct pathological entities and remissions can be achieved with single drug or combined chemotherapy. However, median survival reaches only 5–10 years (Lister et al, 1978; Rosenberg, 1985). The majority of patients present with advanced disease, and therapeutic strategies may vary from watchful waiting to immediate aggressive therapies (Young et al, 1988). Standard treatment is based on an alkylating agent such as cyclophosphamide or chlorambucil, with responses observed in two-thirds of untreated patients. At relapse or in resistant disease, combination regimens may induce some durable responses (Velasquez et al, 1988).

Among agents recently introduced in clinical practice, purine analogues such as cladribine have shown useful activity in both previously treated and untreated patients with indolent lymphoid malignancies (Piro et al, 1990; Beutler, 1992; Kay et al, 1992; Saven et al, 1992; Dimopoulos et al, 1993; Hickish et al, 1993; Juliusson and Liliemark, 1993, 1996; Hoffman et al, 1994; Saven and Piro, 1994; Delannoy et al, 1995; Saven et al, 1995; Betticher et al, 1996). Lack of cross-resistance between cladribine and fludarabine, another purine analogue, has also been reported but not confirmed (Juliusson et al, 1992; O'Brien et al, 1994).

*Received 20 April 1998*
*Revised 14 July 1998*
*Accepted 27 July 1998*

*Correspondence to:* GB Zulian, Cesco, Geneva University Hospitals, CH-1245 Collonge-Bellerive, Switzerland

Inhibition of DNA repair together with the role of the enzymes 5′-nucleotidase and deoxycytidine kinase has been well underlined in determining the activity of the purine analogues (Danhauser et al, 1986; Kawasaki et al, 1993; Robertson et al, 1993). In addition, synergy with other agents, such as the alkylating agents, is expected from in vitro studies and has already been demonstrated in the clinics with fludarabine combined with cyclophosphamide (Hosek et al, 1991; O'Brien et al, 1996).

Cladribine administered as a single agent for 7 days to patients with indolent lymphoid malignancies resulted in dose-dependent infections and cumulative myelotoxicity limited the dose (Betticher et al, 1993, 1994). The same range of complications has been shown with cladribine given in combination with either chlorambucil or mitoxantrone to patients with indolent lymphoid malignancies (Tefferi et al, 1994; Saven et al, 1996). Early enthusiasm for the association of cladribine with other active cytotoxic agents may thus have been hampered by such adverse experiences.

Nevertheless, the rationale still persists for the inclusion of cladribine in the therapeutic armamentarium. Based on our previous data with single-agent cladribine, we have administered this agent in combination with cyclophosphamide and prednisone, the present local standard, to assess the toxicity profile of this combination (CCP) and its activity. Cladribine is registered to be given through a continuous intravenous infusion but this makes the treatment difficult in the outpatient and hospitalization may be necessary. In addition, the bioavailability of subcutaneous cladribine has been shown to be similar to that of the standard intravenous infusion (Liliemark et al, 1992) and we thus selected the subcutaneous route. Oral cladribine could have been an attractive alternative but early results suggested that this route is associated with high and unpredictable toxicity (Juliusson et al, 1996).

### METHODS

### Patients (Table 1)

Between October 1993 and September 1995, patients presenting with CLL Binet stage B–C, IWF NHL-A or M stage III–IV were recruited. Only those not previously treated with purine analogues, a WHO performance ≤2 and a negative HIV serology could enter the study. Diagnosis was established by both cytological and immunological studies of lymph nodes, blood smears and bone marrow aspirate and biopsy. The study was approved by the ethics committee of the department of medicine of Geneva University Hospitals and all patients gave written informed consent.

Pretreatment evaluation included medical history, physical examination, full blood counts with differential and platelets, chemistry profile, chest radiography and abdominal computerized tomography (CT) or ultrasound.

Of the 19 patients included in the study, 11 were men. Median age was 61 years (range 48–86). Nine patients had CLL (two Binet stage B, seven stage C), seven NHL-A (six stage IV, one stage III) and three M. Eight were previously treated (one splenectomy, three chemotherapy and 4, both). Previous chemotherapy consisted in chlorambucil + prednisone in three patients and various combination regimen in four patients (details given in Table 1). Six patients with CLL, 4 with NHL-A and 1 with M were untreated.

**Table 1** Patients' characteristics

| | |
|---|---|
| Age [median (range)] | 61 years (48–86) |
| Sex (Male/female) | 11/8 |
| *Previous treatment* | |
| None | 11 |
| Splenectomy | 5 |
| One year CLB-P | 1 |
| Four months CLB-P | 1 |
| Four months CLB-P, then 16 months IFN + P | 1 |
| Six courses of CVP | 1 |
| One course of CVP + HDS | 1 |
| Six courses of CHOP | 1 |
| One course of CHOP, then six courses of PACE | 1 |
| *Diagnosis* | |
| CLL | 9 |
| NHL-A | 7 |
| M | 3 |

CLL, chronic lymphocytic leukaemia; NHL-A, non-Hodgkin's lymphoma CLL-like; M, macroglobulinaemia; CLB-P, chlorambucil, prednisone; IFN, interferon alpha; CVP, cyclophosphamide, vincristine, prednisone; HDS, high-dose steroids; CHOP, cyclophosphamide, doxorubicin, vincristine, prednisone; PACE, procarbazine, cytarabine, etoposide, cisplatin.

### Treatment (Table 2)

Cladribine was supplied by the Swiss Group for Cancer Clinical Research (SAKK) as a 0.1% solution of 1 mg $ml^{-1}$ pyrogen-free cladribine in sterile 0.9% sodium chloride. Cyclophosphamide and prednisone were purchased from commercial sources. Cladribine 0.1 mg $kg^{-1}$ per day was given subcutaneously for three consecutive days with 500 mg $m^{-2}$ i.v. cyclophosphamide on day 1 and oral prednisone 40 mg $m^{-2}$ $day^{-1}$ on days 1–5. Courses were given every 28 days. In the absence of WHO grade 4 haematological toxicity and grade 3 non-haematological toxicity during courses 1 and 2, cladribine could be increased to 4 days for courses 3 and 4, and up to a maximum of 5 days during courses 5 and 6. Patients and treating physicians were allowed to stop CCP at any time.

Patients attended the clinic once weekly, with a full blood count and other tests carried out as appropriate. On day 29, if leucocytes were $< 3 \times 10^9$ $l^{-1}$ or neutrophils $< 1 \times 10^9$ $l^{-1}$ or thrombocytes $< 100 \times 10^9$ $l^{-1}$, the treatment was delayed for 1 week. In case of no recovery of these counts, the treatment was stopped unless low counts were clearly related to the lymphoid malignancy. A maximum of six courses of chemotherapy was planned. No restriction were made on radiation therapy. Irradiated red blood cells or platelets were given whenever indicated (Zulian et al, 1995). Prophylactic antibiotics and haematopoietic growth factors were not used.

Side-effects were considered from day 1 of the first course until 4 weeks after the end of the last course. Complications were recorded as any events occurring later than one month after CCP until the most recent follow-up.

**Table 2** CCP combination (every 28 days)

| | Day | | | | |
|---|---|---|---|---|---|
| | 1 | 2 | 3 | 4 | 5 |
| Cladribine mg $kg^{-1}$ s.c. | 0.1 | 0.1 | 0.1 | (0.1)[a] | (0.1)[a] |
| Cyclophosphamide mg $m^{-2}$ i.v. | 500 | | | | |
| Prednisone mg $m^{-2}$ p.o. | 40 | 40 | 40 | 40 | 40 |

[a]In the case of < WHO grade 4 haematological toxicity and of < WHO grade 3 non-haematological toxicity.

### Response criteria

Complete response (CR) was defined as the absence of active disease on physical examination, chest radiography and abdominal CT scan or ultrasound, peripheral blood count, bone marrow biopsy and/or aspirate as well as protein electrophoresis. Partial response (PR) was defined as a reduction of > 50% of all measurable disease lasting for > 1 month. Anything else was qualified as no response or progressive disease. Relapse was defined as an increase in serum monoclonal protein by at least 25% in the case of M and reappearance of adenopathy or lymphocytosis $> 5 \times 10^9$ $l^{-1}$ in the case of NHL-A and CLL respectively.

Response was assessed after each cycle and after the third course of CCP at known sites of disease. Response duration was calculated from the first day of the response assessment. In the case of CR, no further therapy was administered and, in the case of PR, CCP was continued up to a maximum of six courses. patients whose disease progressed during CCP could be switched to another treatment.

Follow-up was performed every month for the first trimester after therapy, then every 3 months for 18 months, and thereafter every 6 months until disease progression. Clinical examination was done on each occasion together with full blood counts and chemistry. Other tests were requested based on clinical grounds. Survival was calculated from day 1 of the treatment according to Kaplan and Meier (1958).

### RESULTS

### Dose modification

A total of 80 courses of CCP were administered. One patient received only one 3-day course and declined further treatment. Three patients received two courses, four patients received three

© Cancer Research Campaign 1999

**Table 3** WHO overall toxicity on treatment

| Days of 2CDA | Number of cycles | Course | WBC | | Neutrophils | | | Platelets | Infections | |
|---|---|---|---|---|---|---|---|---|---|---|
| | | | G2 | G3 | G2 | G3 | G4 | G2 | G2 | G3 |
| 3 | 19 | **1** | 1 | 1 | 2 | | | 1 | 1[a] | |
| 3 | 9 | | | | | | | | | |
| 4 | 9 | **2** | 1 | 3 | 1 | 4 | | 1 | | |
| 3 | 2 | | | | | | | | | |
| 4 | 11 | **3** | 3 | 2 | 4 | 2 | 1 | | 1[b] | |
| 5 | 2 | | | | | | | | | |
| 3 | 1 | | | | | | | | | |
| 4 | 8 | **4** | 2 | 1 | 1 | | 1 | | 1[b] | |
| 5 | 2 | | | | | | | | | |
| 4 | 3 | | | | | | | | | |
| 5 | 6 | **5** | 1 | | 1 | | | 1 | | |
| 4 | 3 | | | | | | | | | |
| 5 | 5 | **6** | 3 | 1 | 1 | | | 1 | 1[a] | 1[a] |
| Total | 80 | 80 | 11 | 8 | 10 | 6 | 2 | 4 | 4 | 1 |

[a]Non-viral infection. [b]Herpes (1 HSV2, 1 zoster).

**Table 4** Major haematological and non-haematological toxicities during the follow-up after the completion of treatment

| | Grade 2 | Grade 3 | Grade 4 | Others |
|---|---|---|---|---|
| All toxicities | 15 | 8 | 2 | 1 |
| Anaemia | 1 | 1 | 1 | |
| Thrombopenia | 1 | 1 | | |
| Leucopenia | 3 | 2 | | |
| Neutropenia | 3 | | | |
| Bacterial infection | 4 | | 1 + 1[a] | |
| Herpes | | 4 | | |
| Candidosis | 1 | | | |
| Diarrhoea | 1 | | | |
| Pulmonary | | | 1[a] | |
| Deep venous thrombosis | | | | 1 |
| Neurological toxicity | 1 | | | |

[a]Same patient.

courses, two patients received four courses, one patient received five courses and eight patients received the maximum of six courses. There were 31 cycles of CCP with cladribine given for 3 days, 34 cycles with cladribine for 4 days and 15 cycles with cladribine for 5 days.

### **Haematological toxicity during treatment** (Table 3)

Myelosuppression was the most common side-effect, with 11 episodes of WHO grade 2 leucopenia (15% of cycles), eight grade 3 (10%), ten neutropenia grade 2 (13%), six grade 3 (4%), two grade 4 (2.5%), four thrombocytopenia grade 2 (5%) and no other grade 3 and 4 haematological toxicities.

### **Non-haematological toxicity during treatment** (Table 3)

Nausea, vomiting, alopecia, mucositis, hepatic or renal failure and neurological toxicity were non-existent. WHO grade 3 lung infection occurred in one patient during the sixth course of CCP, requiring hospitalization and parenteral antibiotics. WHO grade 2 lung infection occurred in one patient each during courses one, four, five and six. Identification of the responsible pathogen was unsuccessful in every case. There were no treatment-related deaths.

### **Haematological complications after treatment** (Table 4)

There were eight WHO grade 2 haematological toxicities, four grade 3 and one grade 4. Mean lymphocyte count 1 month after the end of CCP was 735 G $l^{-1}$ (median 759 G $l^{-1}$, 350–6850); 1404 G $l^{-1}$ after 3 months (median 1450 G $l^{-1}$, 218–1700); 1565 G $l^{-1}$ after 6 months (median 4000 G $l^{-1}$, 100–70 870) and 2549 G $l^{-1}$ after 9 months (median 6800 G $l^{-1}$, 351–72 944).

### **Non-haematological complications after treatment** (Table 4)

There were 15 non-haematological complications, seven WHO grade 2, four grade 3, two grade 4 and one non-gradable deep vein thrombosis. Among these patients, one developed reversible adult respiratory distress syndrome as a result of pneumococcaemia 13 months after the end of CCP. One patient died as the result of septicaemia due to *Streptococcus pneumoniae* 17 months after the end of CCP.

### **Response**

One patient was not assessable for response because he declined further treatment on day 3 of the first course. Eighteen patients are thus evaluable for response. After three courses, one patient achieved CR and 15 achieved PR for an overall response rate of 88%. One pretreated CLL patient progressed and one untreated NHL had no response. Three patients in PR after three courses further responded to achieve CR after six courses. Overall CR rate is thus 22% and 12 patients achieved PR for an unchanged overall response rate of 88%. CR was obtained in two patients with untreated CLL, one with previously treated CLL and one with previously treated NHL-A. PR was obtained in three patients with M, two patients with previously treated CLL, three with previously untreated CLL, three with untreated NHL and two with pretreated NHL-A. Four patients received 30 Gy involved fields radiation therapy in CR or in PR.

### *Relapses, progressions and survival*

Eleven patients have relapsed, including four patients in CR, and six have progressed from PR. After a minimum follow-up of 20 months, median duration of CR is 12 months and median duration of PR is 15 months. Nine relapsing patients received further treatment, four with purine analogues, two with prednisone and chlorambucil, one with cyclophosphamide, two with radiotherapy and two had a splenectomy. Overall survival is 68% at 3 years (95% confidence interval, 36–85%). Six patients have died with PD.

### DISCUSSION

Alkylating agents such as cyclophosphamide and chlorambucil with or without the association of prednisone are considered the standard cytotoxic treatment of CLL, NHL-A and M. Despite remission rates as high as 70%, no cure is expected and the median survival of 7 years does not make this treatment optimal. In addition, aggressive combination chemotherapy has not improved the outlook for the majority of patients (French Cooperative Group on Chronic Lymphocytic Leukemia, 1986).

In the present study of a mixed group of patients, we have shown the feasibility of combining the purine analogue cladribine with the standard treatment of cyclophosphamide and prednisone. In contrast to other studies using cladribine in association with either chlorambucil or mitoxantrone (Tefferi et al, 1994; Saven et al, 1996), the infection rate of our CCP combination was acceptable. This may indeed be attributable to selection bias as over 50% of the patients were previously untreated and could have better tolerated the treatment. Another explanation is the difference in the doses of cladribine that were given. In our study, cladribine was administered for 3 days during the first course as opposed to 7 days in the other studies. Furthermore, a maximum of 5 days was allowed, resulting in a lower total dose for the patients completing the whole treatment.

The first three courses of the CCP combination were associated with moderate myelosuppression and few infections. The total amount of cladribine at that point of the treatment was relatively low because patients received a maximum of 4 days of cladribine during the first three courses. This is the most likely explanation for the absence of detrimental toxicity. In addition, the majority of responses were already achieved by that time and some patients stopped their treatment. This is perhaps an indication that patients responding quickly to CCP should be spared additional courses in order to avoid potential cumulative toxicity.

Significant neutropenia or thrombocytopenia occurred only in those patients continuing on the protocol to receive the maximum of 5 days of cladribine. All patients eventually recovered from toxicity, and no treatment-related death occurred. The overall remission rate was not modified by additional courses of CCP, but two patients in PR converted into CR indicating the continuous activity of this combination in some cases. However, no attempt to further escalate cladribine to the maximum tolerated dose was contemplated in the present study.

Overall, the CCP combination was subjectively well tolerated and could always be given in the outpatient facilities. Only one hospitalization was necessary while the patient was on treatment. We thus consider CCP to be cost-effective.

During the follow-up after CCP, two major life-threatening bacterial infectious complications occurred, confirming a sustained immunosuppression (Seymour et al, 1994; Cheson, 1995). This should further point out our difficulties in selecting a treatment after exposure to purine analogues and the need for alternatives.

On the basis of these observations, the combination of cladribine at 0.1 mg $kg^{-1}$ per day for 3 days with standard dose of cyclophosphamide and prednisone appears safe in this small group of patients. It remains to be seen whether under certain circumstances, such as perhaps in selected untreated patients, cladribine could be administered for longer together with the same combination of cyclophosphamide and prednisone, and this is presently under study. A further step would then require the randomized comparison of adding cladribine to the combination of cyclophosphamide and prednisone.

### REFERENCES

Betticher DC, Fey MF, Rabaglio M, Cerny T, Hess U, Meier V, Stalder M and Zulian G (1993) Cladribine and severe myelotoxicity. *Lancet* **342**: 1369

Betticher DC, Fey MF, von Rohr A, Tobler A, Jenzer H, Gratwohl A, Lohri A, Pugin P, Hess U, Pagani O, Zulian G and Cerny T (1994) High incidence of infections after 2-chlorodeoxyadenosine (2-CdA) therapy in patients with malignant lymphomas and chronic and acute leukaemias. *Ann Oncol* **5**: 57–64

Betticher DC, Zucca E, von Rohr A, Egger T, Radford JA, Ambrosetti A, Burki K, Rufener B, Schmitz SF and Cerny T (1996) 2-Chlorodeoxyadenosine (2CdA) therapy in previously untreated patients with follicular stage III–IV non-Hodgkin's lymphoma. *Ann Oncol* **7**: 793–799

Beutler, E (1992) Cladribine. *Lancet* **340**: 952–956

Cheson BD (1995) Infectious and immunosuppressive complications of purine analog therapy. *J Clin Oncol* **13**: 2431–2448

Danhauser L, Plunkett W, Keating M and Cabanillas F (1986) 9-β-D-arabinofuranosyl-2-fluoro-adenine 5′-monophosphate pharmocokinetics in plasma and tumor cells of patients with relapsed leukemia and lymphoma. *Cancer Chemother Pharmacol* **18**: 145–152

Delannoy A, Martiat P, Gala JL, Deneys V, Ferrant A, Bosly A, Scheiff JM and Michaux JL (1995) 2-Chlorodeoxyadenosine (CdA) for patients with previously untreated chronic lymphocytic leukemia (CLL). *Leukemia* **9**: 1130–1135

Dimopoulos MA, Kantarjian H, Estey E, O'Brien S, Estex E, Delasalle K, Keating MJ, Freireich EJ & Alexanian R (1993) Treatment of Waldenstrom macroglobulinemia with 2-chlorodeoxyadenosine. *Ann Intern Med* **118**: 195–198

French cooperative group on chronic lymphocytic leukemia (1986) Effectiveness of CHOP regimen in advanced untreated chronic lymphocytic leukemia. *Lancet* **327**: 1346–1349

Hickish T, Serafinowski P, Cunningham D, Oza A, Dorland E, Judson I, Millar BC, Lister TA and Roldan A (1993) 2′-chlorodeoxyadenosine: evaluation of a novel predominantly lymphocyte selective agent in lymphoid malignancies. *Br J Cancer* **67**: 139–143

Hoffman M, Tallman MS, Hakimian D, Janson D, Hogan D, Variakogis D, Kuzel T, Gordon LI and Rai K (1994) 2-chlorodeoxyadenosine is an active salvage therapy in advanced indolent non-Hodgkin's lymphoma. *J Clin Oncol* **12**: 788–792

Hosek B, Bohacek J and Sikulova J (1991) The effect of cyclophosphamide and gamma irradiation on adenosine deaminase and purine nucleoside phosphorylase in mice. *Life Sci* **49**: 1403–1407

Juliusson G and Liliemark J (1993) High complete remission rate from 2-chloro-2′-deoxyadenosine in previously treated patients with chronic lymphocytic leukemia: response predicted by rapid decrease of blood lymphocyte count. *J Clin Oncol* **11**: 679–689

Juliusson G and Liliemark J (1996) Long term survival following cladribine (2-chlorodeoxyadenosine) therapy in previously treated patients with chronic lymphocytic leukemia. *Ann Oncol* **7**: 373–379

Juliusson G, Elmhorn-Rosenborg A and Liliemark J (1992). Response to 2-chlorodeoxyadenosine in patients with B-cell chronic lymphocytic leukemia resistant to fludarabine. *N Engl J Med* **327**: 1056–1061

 *© Cancer Research Campaign 1999*

Juliusson G, Christiansen I, Hansen MM, Johnson S, Kimby E, Elmhorn-Rosenborg A and Liliemark J (1996) Oral cladribine as primary therapy for patients with B-cell chronic lymphocytic leukemia. *J Clin Oncol* **14**: 2160–2166

Kaplan HS and Meier P (1958) Nonparametric estimation from incomplete observations. *Am J Stat Assoc* **53**: 457–481

Kawasaki H, Carrera CJ, Piro LD, Saven A, Kipps TJ and Carson DA (1993) Relationship of deoxycytidine kinase and cytoplasmic 5′-nucleotidase to the chemotherapeutic efficacy of 2-chlorodeoxyadenosine. *Blood* **81**: 597–601

Kay AC, Saven A, Carrerra CJ, Carson DA, Beutler E and Piro LD (1992) 2-chlorodeoxyadenosine in low grade lymphoma. *J Clin Oncol* **10**: 371–377

Liliemark J, Albertioni F, Hassan M and Juliusson G (1992) On the bioavailability of oral and subcutaneous 2-chloro-2′-deoxyadenosine in humans: alternative routes of administration. *J Clin Oncol* **10**: 1514–1518

Lister T, Cullen MH, Beard ME, Brearley RL, Whitehouse JM, Wrigley PF, Stansfeld AG, Sutcliffe SB, Malpas JS and Crowther D (1978) Comparison of combined and single-agent chemotherapy in non-Hodgkin's lymphoma of favorable histological type. *Br Med J* **1**: 533–537

Nagourney RA, Evans SS, Messenger JC, Zhuang Su, Y and Weisenthal LM (1993) 2-chlorodeoxyadenosine activity and cross resistance patterns in primary cultures of human hematologic neoplasms. *Br J Cancer* **67**: 10–14

O'Brien S, Kantarjian H, Estey E, Koller C, Robertson B, Beran M, Andreef M, Pierce S and Keating M (1994) Lack of effect of 2-chlorodeoxyadenosine therapy in patients with chronic lymphocytic leukemia refractory to fludarabine. *N Engl J Med* **330**: 319–322

O'Brien S, Kantarjian H and Keating M (1996) Purine analogs in chronic lymphocytic leukemia and Waldenström macroglobulinemia. *Ann Oncol* **7**(suppl. 6): 27–33

Piro LD, Carrera CJ, Carson DA and Beutler E (1990) Lasting remissions in hairy-cell leukemia induced by a single infusion of 2-chlorodeoxyadenosine. *N Engl J Med* **322**: 1117–1121

Robertson LE, Chubb S, Meyn RE, Story M, Ford R, Hittelman WN and Plunkett W (1993) Induction of apoptotic cell death in chronic lymphocytic leukemia by 2-chloro-2′-deoxyadenosine and 9-β-d-arabinosyl-2-fluoroadenine. *Blood* **81**: 143–150

Rosenberg AS (1985) The low-grade non-Hodgkin's lymphomas: challenges and opportunities. *J Clin Oncol* **3**: 299–310

Saven A and Piro LD (1994) 2-chlorodeoxyadenosine: a newer purine analog active in the treatment of indolent lymphoid malignancies. *Ann Intern Med* **120**: 784–791

Saven A, Carrera CJ, Carson DA, Beutler E and Piro LD (1992) 2-chloro-deoxyadenosine: an active agent in the treatment of cutaneous T-cell lymphoma. *Blood* **80**: 587–592

Saven A, Emanuele S, Kosty M, Koziol J, Ellison D and Piro L (1995) 2-chloro-deoxyadenosine activity in patients with untreated indolent non-Hodgkin's lymphoma. *Blood* **86**: 1710–1716

Saven A, Lee T, Kostly M and Piro L (1996) Cladribine and mitoxantrone dose escalation in indolent non-Hodgkin's lymphoma. *J Clin Oncol* **14**: 2139–2144

Seymour JF, Kurzrock R, Freireich EJ and Estey EH (1994) 2-chlorodeoxyadenosine induces durable remissions and prolonged suppression of CD4+ lymphocyte counts in patients with hairy cell leukemia. *Blood* **83**: 2906–2911

Tefferi A, Witzig TE, Reid JM, Chin-Yiang L and Ames MM (1994) Phase I study of combined 2-chlorodeoxyadenosine and chlorambucil in chronic lymphocytic leukemia and low-grade lymphoma. *J Clin Oncol* **12**: 569–574

Velasquez WS, Cabanillas F, Salvador P, McLaughlin P, Fridrik M, Tucker S, Jagannath S, Hagemesiter FB, Redman JR, Swan F and Barlogie B (1988) Effective salvage therapy for lymphoma with cisplatine in combination with high-dose ara-C and dexamethasone (DHAP). *Blood* **71**: 117–122

Young R, Longo D, Glatstein E, Ihde DC, Jaffe ES and De Vita VJ (1988) The treatment of indolent lymphomas; watchful waiting versus aggressive combined modality treatment. *Sem Hematol* **25**: 11–16

Zulian GB, Roux E, Tiercy JM, Extermann M, Diebold-Berger S, Reymond JM, Helg C, Zubler R, Betticher DC, Alberto P, Jeannet M and Chapuis B (1995) Transfusion associated graft-versus-host disease in a patient treated with cladribine (2-chlorodeoxyadenosine): demonstration of exogenous DNA in various tissue extracts by PCR analysis. *Br J Haematol* **89**: 83–89

*© Cancer Research Campaign 1999*
